# No evidence for a diminished ovarian reserve among patients with hypertensive disorders of pregnancy: a case control study

**DOI:** 10.1186/s13048-023-01333-9

**Published:** 2024-01-06

**Authors:** Bo E. van Bree, Laura M. Jorissen, Désirée A.P.M. Pattinaja, Judith A.P. Bons, Marc E.A. Spaanderman, Olivier Valkenburg, Ron J.T. van Golde

**Affiliations:** 1 Department of Obstetrics & Gynaecology, Maastricht University Medical Centre+, 5800, 6202 AZ Maastricht, The Netherlands; 2 GROW School for Oncology and Reproduction, Maastricht University, Maastricht, The Netherlands; 3 Central Diagnostic Laboratory, Maastricht University Medical Centre+, Maastricht, The Netherlands

**Keywords:** Ovarian reserve, Anti-Müllerian hormone, Hypertensive disorders of pregnancy, Preeclampsia, Gestational Hypertension, Cardiovascular dysfunction

## Abstract

**Background:**

Existing evidence suggests a relation between cardiovascular dysfunction and diminished ovarian reserve. While it is known that pre-existent cardiovascular dysfunction is also associated with the development of preeclampsia (PE) during pregnancy, we hypothesize that signs of diminished ovarian reserve may occur more frequently among women with a history of hypertensive disorders of pregnancy (HDP). The aim of our study was therefore to analyse if women with a history of HDP show signs of diminished ovarian reserve, represented by lower anti-Mullarian hormone (AMH) levels, compared to controls. For this retrospective observational case control study, patients included women with a history of HDP, whereas controls constituted of women with a history of an uncomplicated pregnancy. The study was conducted in a tertiary referral centre in which all women underwent a one-time cardiovascular and metabolic assessment. Ovarian reserve and markers of cardiovascular function were evaluated, adjusted for age and body mass index (BMI) using linear regression analyses.

**Results:**

163 patients and 81 controls were included over a time span of 3 years. No signs of diminished ovarian reserve i.e. lower AMH level were observed in the patient group versus controls. A subgroup analysis even showed higher AMH levels in late onset HDP as compared to controls (2.8 vs. 2.0 µg/L, *p* = 0.025). As expected, cardiovascular function markers were significantly less favourable in the patient group compared to controls; higher levels of systolic blood pressure (BP) (5%), diastolic BP (4%), triglycerides (29%), glucose (4%) and insulin levels (81%) (all *p* < 0.05), whereas high density lipid (HDL) cholesterol was 12% lower (NS).

**Conclusions:**

Despite unfavourable cardiovascular risk profile, the present study does not substantiate the hypothesis that women with HDP show accelerated ovarian ageing as compared to healthy parous controls. Although HDP patients should be warned about their cardiovascular health, they shouldn’t be concerned about unfavourable ovarian reserve status.

**Supplementary Information:**

The online version contains supplementary material available at 10.1186/s13048-023-01333-9.

## Background

Prior research showed that decreased ovarian reserve, represented by low oocyte yield during in-vitro fertilization (IVF) treatment or low Anti-Müllerian hormone (AMH), could be associated with an increased risk of developing preeclampsia (PE) during pregnancy [1]. PE, classically defined by hypertension and proteinuria after the 20th week of pregnancy, relates to foetal and maternal morbidity and mortality in 2–8% of all pregnancies [2, 3]. In line with these findings, Yarde et al. observed 10% lower AMH among women with a history of PE [4]. These findings may suggest an association between decreased ovarian reserve and PE, which may have consequences for future reproductive plans and preventive care. However, this association has not been confirmed to date.

Serum AMH levels show a consistent decline that correlates with the age dependent depletion of the primordial and antral follicle pool [5]. Furthermore, it was also shown that the decline of AMH levels is independently and inversely associated with the risk of cardiovascular disease (CVD) [6], even when adjusted for age. Therefore, it can be hypothesised that AMH reflects cardiovascular status in women of childbearing age. As such, women with low AMH levels during pregnancy show an increased risk of gestational hypertension and CVD markers [7, 8] as well as other hypertensive disorders of pregnancy (HDP), placental malperfusion lesions and miscarriage [9–11]. Decreased ovarian reserve is also associated with a lower rate of pregnancy and live birth [12]. Women with a history of PE are at risk for CVD, especially with ageing [13]. Interestingly, the prevalence of PE is highly associated with pre-existing attenuated cardiovascular function [14]. Based on this knowledge, we expect that women with a history of any HDP show a less favourable cardiovascular status.

The possible link between cardiovascular health and ovarian function can be studied from a different perspective as well. A genome-wide association study for age at natural menopause identified several common genetic variants that play a role in timing of ovarian aging [15]. Most of these loci appear to be involved in DNA damage response processes [16]. In line with this finding, it is suggested that ovarian ageing and early menopause may result from processes of general ageing, through DNA damage and genetic susceptibility [17].

We speculate that common denominators of cardiovascular health, such as, but not limited to, genetic variance and/or cardiovascular dysfunction may play a role in the predisposition for both HDP and ovarian reserve capacity. This can be further substantiated by the observation that the ovaries are highly vascularized organs, so impaired blood flow can induce oxidative stress and thereby increase follicular demise [4]. It is expected that different rates of ovarian follicle pool depletion can contribute to wide variation in fecundity among fertile women [18, 19]. Unravelling underlying mechanisms may increase our understanding of reproductive health and lifespan.

We hypothesize that there is a concomitant association between lower AMH levels—reflecting a diminished ovarian reserve—and a higher rate of cardiovascular dysfunction is observed in women with a history of HDP. The aim of this study is to evaluate cardiovascular function and ovarian reserve among women with a history of HDP and healthy controls.

## Results

### Study population, baseline characteristics

163 patients and 81 controls were included. There was a small but significant difference in age between patients and controls (31.7 vs. 34.1 years, respectively, *p* < 0.001). We observed 6.9% higher body weight (*p* = 0.002) and 9.3% higher BMI (*p* < 0.001) among patients. Parity was significantly lower for patients (1 vs. 2, *p* < 0.001). As expected, duration of pregnancy was significantly shorter in patients (241 vs. 279 days, *p* < 0.001) and their children had lower birth weight (2105 vs. 3432 g, *p* < 0.001). Controls were significantly longer postpartum at time of investigation (191 vs. 74 weeks, *p* < 0.001). Baseline characteristics are summarized in Table 1.

**Table 1:** Baseline characteristics and obstetric history

	Patients n = 163	Controls n = 81	p-value patients vs. controls
**Age (years)**	31.7 ± 4.1	34.1 ± 4.7	< 0.001*
**Height (meters)**	1.68 ± 0.07	1.70 ± 0.08	0.097
**Weight (kilograms)**	73.2 ± 14.3	68.5 ± 14.3	0.002*
**BMI (kilograms/meter** ^**2**^ **)**	25.9 ± 5.0	23.7 ± 4.6	< 0.001*
**Gravidity (n)**	1 [1–9]	2 [1–4]	< 0.001*
**Parity (n)**	1 [1–3]	2 [1–4]	< 0.001*
**Birthweight (grams)**	2105 ± 903	3432 ± 481	< 0.001*
**Gestational age at delivery (days)**	241 ± 29	279 ± 9	< 0.001*
**Time postpartum (weeks)**	74 ± 71	191 ± 149	< 0.001*

Data are presented as mean ± standard deviation or as median [range]

* statistically significant

### Ovarian reserve and cardiovascular function

Ovarian reserve, represented by AMH, was 20% higher among patients compared to controls (2.4 vs. 2.0 µg/L). This difference remained significant after correction for age (*p* = 0.045). There was a significant inverse relation between age and AMH (B=–0.143, *p* = 0.002). No significant correlations between AMH and BMI or other cardiovascular function parameters were found. Cardiovascular function parameters showed less favourable results among patients; higher fasting glucose (4%, *p* = 0.016), insulin (81%, *p* < 0.001), systolic BP (5%, *p* = 0.001), diastolic BP (4%, *p* < 0.001), MAP (6%, *p* < 0.001) and triglycerides (29%, *p* = 0.002). Ovarian reserve and cardiovascular function are summarized in Table 2.

**Table 2:** Ovarian reserve and cardiovascular function, corrected for age and BMI

	Patients n = 163	Controls n = 81	p-value patients vs. controls
**AMH level (µg/L)**	2.4 [1.2–4.5]	2.0 [0.7–3.5]	0.045*
**Glucose (mmol/L)**	5.0 [4.8–5.2]	4.8 [4.6–5.1]	0.016*
**Insulin (mU/L)**	7.8 [5.3–11.3]	4.3 [2.8–6.9]	< 0.001*
**Systolic BP (mmHg)**	113 [108–121]	108 [103–114]	0.001*
**Diastolic BP (mmHg)**	72 [67–76]	69 [63–75]	< 0.001*
**MAP (mmHg)**	88 [83–94]	83 [78–89]	< 0.001*
**Total cholesterol (mmol/L)**	4.5 [4.1–5.1]	4.5 [4.1–4.9]	0.305
**HDL cholesterol (mmol/L)**	1.4 [1.2–1.7]	1.6 [1.3–1.8]	0.231
**Triglycerides (mmol/L)**	0.9 [0.7–1.2]	0.7 [0.6–0.9]	0.002*

Data are presented as median [IQR]. All data are corrected for age and BMI

* statistically significant

A correlation matrix of AMH and age, blood pressure and BMI is shown in Appendix 1, to show the associations between these measurements.

### Subgroup analysis

For the subgroup analysis, patients were divided in early (< 34 weeks) and late (≥ 34 weeks) onset of HDP. In comparison to controls, significant differences in age, weight, BMI, gravidity, parity, birth weight, gestational age at delivery and time postpartum at measurement were observed in both subgroups. No difference in AMH was observed in the early onset group compared to controls. However, significant higher AMH levels were observed in the late onset group, compared to controls (2.8 vs. 2.0 µg/L, *p* = 0.025).

In the early onset group, significantly higher fasting glucose levels (4%, *p* = 0.014) than controls were observed. Both subgroups showed comparable differences with higher insulin (98% in early and 70% in late onset, respectively, *p* < 0.001), systolic BP (6% and 5%, *p* < 0.05), diastolic BP (4% and 3%, *p* < 0.01), MAP (6% and 5%, *p* = 0.004) and triglycerides (both 29%, *p* < 0.05) than controls.

The comparison between the subgroups shows significantly higher BMI and borderline lower triglycerides in the early onset group. Birth weight and gestational age at delivery were lower among early onset cases, as could be expected. Results of the subgroup analysis are summarized in Table 3.

**Table 3:** Subgroup analysis of early and late onset HDP, corrected for age and BMI

	Controls n = 81	Early onset HDP < 34 weeks n = 83	p-value < 34 weeks vs controls	Late onset HDP ≥ 34 weeks n = 61	p-value ≥ 34 weeks vs controls	p-value < 34 vs ≥ 34 weeks
**Age (years)**	35 [30–38]	32 [29–35]	0.001*	31 [29–34]	< 0.001*	0.570
**Height (meters)**	1.69 [1.65–1.74]	1.68 [1.62–1.73]	0.076	1.69 [1.65–1.73]	0.408	0.365
**Weight (kilograms)**	65.0 [59.0–74.0]	77.0 [66.0–85.0]	< 0.001*	68.0 [61.5–76.0]	0.284	0.001*
**BMI (kilogram/meter** ^**2**^ **)**	23.0 [21.0-25.3]	26.4 [23.7–30.8]	< 0.001*	23.6 [21.7–26.7]	0.234	< 0.001*
**Gravidity (n)**	2 (1–4)	1 (1–9)	< 0.001*	1 (1–5)	< 0.001*	0.951
**Parity (n)**	2 (1–4)	1 (1–3)	< 0.001*	1 (1–3)	< 0.001*	0.580
**Birthweight (grams)**	3440 [3027–3780]	1570 [1070–1900]	< 0.001*	2770 [2413–3118]	< 0.001*	< 0.001*
**Gestational age (days)**	280 [273–286]	229 [208–238]	< 0.001*	260 [255–271]	< 0.001*	< 0.001*
**Time postpartum (weeks)**	128 [93–258]	52 [30–92]	< 0.001*	52 [31–86]	< 0.001*	0.963
**AMH level (µg/L)**	2.0 [0.7–3.5]	2.4 [0.9–4.5]	0.215	2.8 [1.5–4.3]	0.025*	0.330
**Glucose (mmol/L)**	4.8 [4.6–5.1]	5.0 [4.8–5.2]	0.014	4.9 [4.8–5.2]	0.103	0.872
**Insulin (mU/L)**	4.3 [2.8–6.9]	8.5 [6.0-14.6]	< 0.001*	7.3 [5.2–9.9]	< 0.001*	0.571
**Systolic BP (mmHg)**	108 [103–114]	114 [109–121]	0.017*	113 [108–120]	0.001*	0.508
**Diastolic BP (mmHg)**	69 [63–75]	72 [68–78]	0.002*	71 [67–75]	0.004*	0.783
**MAP (mmHg)**	83 [78–89]	88 [83–95]	0.004*	87 [82–92]	0.004*	0.954
**Total cholesterol (mmol/L)**	4.5 [4.1–4.9]	4.5 [3.9–5.2]	0.408	4.5 [4.1–5.1]	0.673	0.923
**HDL cholesterol (mmol/L)**	1.6 [1.3–1.8]	1.4 [1.2–1.7]	0.154	1.4 [1.2–5.1]	0.865	0.430
**Triglycerides (mmol/L)**	0.7 [0.6–0.9]	0.9 [0.7–1.2]	0.025*	0.9 [0.7–1.3]	< 0.001*	0.050

Data are presented as median [IQR] or as median (range). All data are corrected for age and BMI

* statistically significant

## Discussion

The present study shows no association between patients with a history of HDP and diminished ovarian reserve. Our findings are in line with a prospective observational study by Bhide et al. who also observed that ovarian reserve parameters were not diminished among women with a history of PE [20]. Another matched control study by van Disseldorp et al. found no significant difference in prevalence of gestational hypertension or PE comparing pregnancies of women with advanced ovarian ageing to pregnant women with normal IVF response [21].

In contrast to our findings, Yarde et al. did observe lower AMH levels in women with a history of PE compared to women with a previous uncomplicated pregnancy [4]. This outcome may be attributed to the fact that AMH levels in that study were measured approximately 10 years after the complicated pregnancy, in contrast to a mean follow up time of 2 years in our study. The mean age of our study population (31.7 ± 4.1 vs. 34.1 ± 4.7) is lower than the study population of Yarde et al. (38.8 ± 4.9 vs. 39.3 ± 4.3). Variable age related decline of AMH during reproductive life span might account for different findings between the studies, even though we did not find a difference in age related decline within our patient population.

Similarly, a retrospective cohort study analysing obstetric outcomes in IVF pregnancies in patients with diminished ovarian reserve observed a higher incidence of PE and placental foetal vascular lesions when compared to controls [22], underlining the conflicting evidence concerning this subject. A possible explanation might be the difference in study design, as Herman et al. specifically observe patients with diminished ovarian reserve according to a cut-off point, in contrast to our study which analyses AMH levels in HDP patients and controls as a continuous outcome measure.

In our study, ovarian reserve was not diminished in HDP patients compared to controls, also after correction for age and BMI. Subgroup analysis showed that specifically late onset HDP was associated with significantly higher AMH levels. This finding emphasizes the possibility that early and late onset HDP have a different aetiology. I.e. early onset HDP patients show higher BMI and might have more cardiovascular damage. One can speculate that late onset HDP patients might be more represented by anovulatory subfertility that is often accompanied by higher AMH levels [23]. While the prevalence of PCOS, time to pregnancy or use of fertility medication is not known in our study it is important to stress that the study was not conducted in a subfertile population.

As expected, there was clear evidence for attenuated cardiovascular function among former HDP patients i.e., higher systolic and diastolic BP, MAP, BMI, triglycerides, glucose and insulin levels and lower HDL cholesterol, indicative for a more atherogenic profile. However, since HDP is known to induce long-term cardiovascular dysfunction, the follow-up time between pregnancy and analysis might influence the cardiovascular parameters as well, especially because lifestyle and environmental factors can be adjusted throughout the years. It is known that higher maternal age increases chance of HDP, however this is only a small attribution and since age is adjusted for, we do not expect this to influence our results.

Strengths of the present study are that all measurements were performed by trained staff with the use of standardized questionnaires and protocols to provide high precision data. Furthermore, all AMH serum samples were analysed in the same ISO accredited laboratory, as were all other laboratory measurements. Limitations include a relatively small patient population, especially in the control group. However, post hoc sample size calculation shows it to be sufficient to detect a 2-years increase of ovarian ageing in patients with a power of 80% and an α of 5%. Next to the limitations stated above, it should be noted that extrapolation of the conclusions of this observational case control study to all subjects of the general population should be done with care.

## Conclusions

In conclusion, the present study shows that a history of HDP is not associated with diminished ovarian reserve, as quantified by lower AMH concentration. Our results do substantiate the close relationship between cardiovascular risk factors and HDP.

## Methods

### Study design

For this retrospective observational case control study with the aim to evaluate cardiovascular function and ovarian reserve among women with a history of HDP and healthy controls, patients aged 18 to 41 years old were recruited at the tertiary outpatient clinic at the Maastricht University Medical Centre+ (MUMC+). They were at least six months postpartum which allows hormonal markers to normalize. All patients had a history of HDP (gestational hypertension or PE), diagnosed according to the criteria specified in 2018 by the International Society for the Study of Hypertension in Pregnancy (ISSHP) [24]. Gestational hypertension is defined as new onset hypertension after 20 weeks of gestation in the absence of proteinuria and without biochemical or haematological abnormalities, with a systolic blood pressure (BP) ≥ 140 mmHg and/or diastolic BP ≥ 90 mmHg. PE is defined as gestational hypertension with proteinuria and/or evidence of maternal acute kidney injury (AKI), liver dysfunction, neurological features, haemolysis or thrombocytopenia, or foetal growth restriction. The control group consisted of healthy women aged 18 to 41 years with a history of an uncomplicated, naturally conceived pregnancy. Controls were recruited through local advertisements and internet. Exclusion criteria for both groups were current pregnancy, breastfeeding, hormonal medication and ovarian surgery.

A post hoc power analysis was conducted using the OpenEpi statistics program. Based the included number of patients and controls, the mean AMH levels and standard deviations in our data and using α of 5%, the actual power is approximated to be 91,14%.

### Measurements

All women underwent cardiovascular and metabolic assessment. Physical examination was performed by a trained research physician, and included measurement of height (meters), body weight (kilograms) and BP (systolic, diastolic and mean arterial pressure (MAP); mmHg). The latter was measured in sitting position after 10 min of rest, every 3 min for a time period of 30 min. The median of eleven consecutive measurements was taken as representative. Body mass index (BMI) was calculated by weight/height^2^ (kg/m^2^).

Fasting blood samples were collected for measurement of AMH, lipid profile, insulin and glucose levels. AMH levels were analysed at the clinical chemical laboratory of the Erasmus MC, Rotterdam, the Netherlands. All samples were stored at -20˚C until assayed. AMH levels were determined by ELISA (GenII Beckman Coulter, Inc., Webster, Texas). For analysis, serum AMH levels below 0.1 µg/L were valued at 0.0. Other measurements were analysed at the central diagnostic laboratory of the MUMC+. Serum total cholesterol, high density lipoprotein (HDL) cholesterol, triglycerides and glucose were analysed using an enzymatic colorimetric assay (Cobas 8000 instrument, Roche Diagnostics, Mannheim, Germany). Serum insulin was determined using a chemiluminescent immunometric assay (XPi instrument, Siemens Medical Solutions Diagnostics, LA).

### Statistical analysis

Data were checked for a normal distribution with Shapiro-Wilk test. Baseline characteristics were summarized as mean and standard deviations and compared using Student t-test. Gravidity and parity were compared using chi-square test. Ovarian reserve and cardiovascular function were summarized as median and interquartile range (IQR). All data were corrected for age and BMI and compared with linear regression analysis. Correlation coefficients between ovarian reserve and cardiovascular function parameters were computed with Spearman’s rho test. Data in the subgroup analysis with early onset (< 34 weeks gestational age) and late onset (≥ 34 weeks gestational age) cases were summarized as median and IQR. Correction for age and BMI was again performed with linear regression analysis. A two-tailed p-value ≤ 0.05 was considered as significant. Statistical analyses were performed using the statistical software SPSS (version 25).

### Electronic supplementary material

Below is the link to the electronic supplementary material.

Supplementary Material 1

## Data Availability

The datasets used and analysed during the current study are available from the corresponding author on reasonable request.

## Abbreviations

AKI: Acute kidney injury
AMH: Anti-Mullarian hormone
BMI: Body mass index
BP: Blood pressure
CI: Confidence interval
CVD: Cardiovascular disease
HDL: High density lipoprotein
HDP: Hypertensive disorders of pregnancy
IQR: Interquartile range
ISSHP: International Society for the Study of Hypertension in Pregnancy
IVF: In-vitro fertilization
MAP: Mean arterial pressure
PCOS: Polycystic ovary syndrome
PE: Preeclampsia
