# Supplementary figures and images for: No evidence for a diminished ovarian reserve among patients with hypertensive disorders of pregnancy: a case control study
Source: J Ovarian Res. 2024 Jan 6;17:5. doi: 10.1186/s13048-023-01333-9 (PMC10770972; doi:10.1186/s13048-023-01333-9)

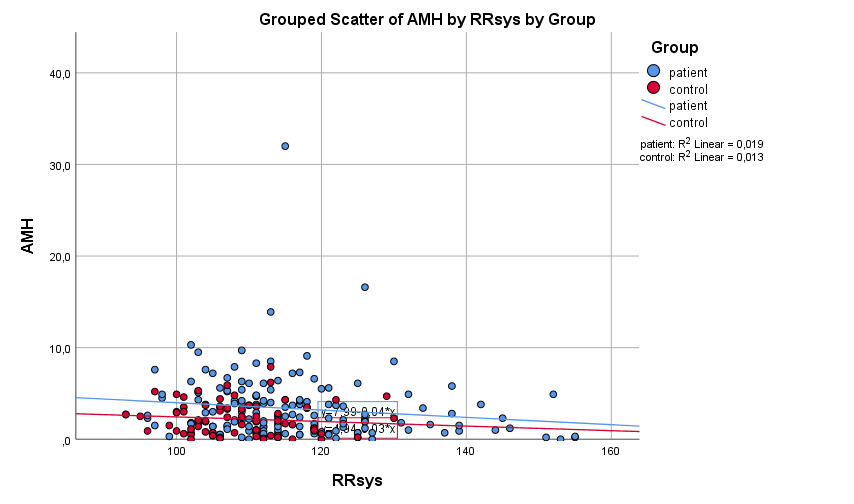

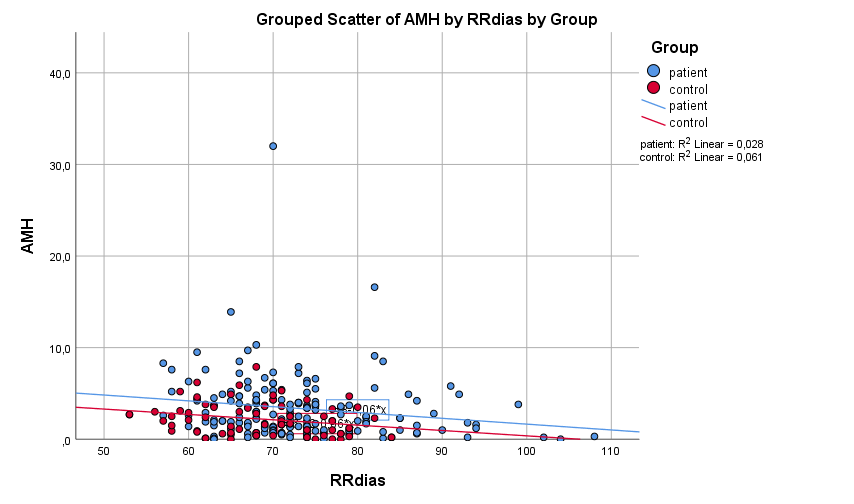

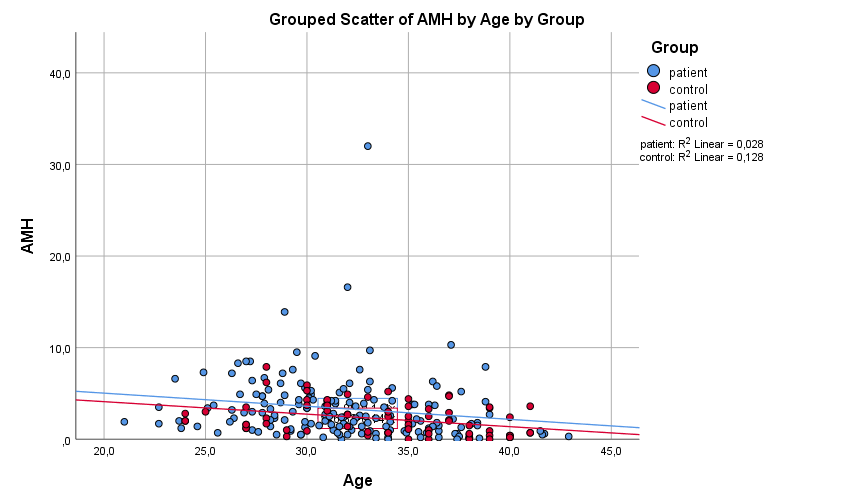

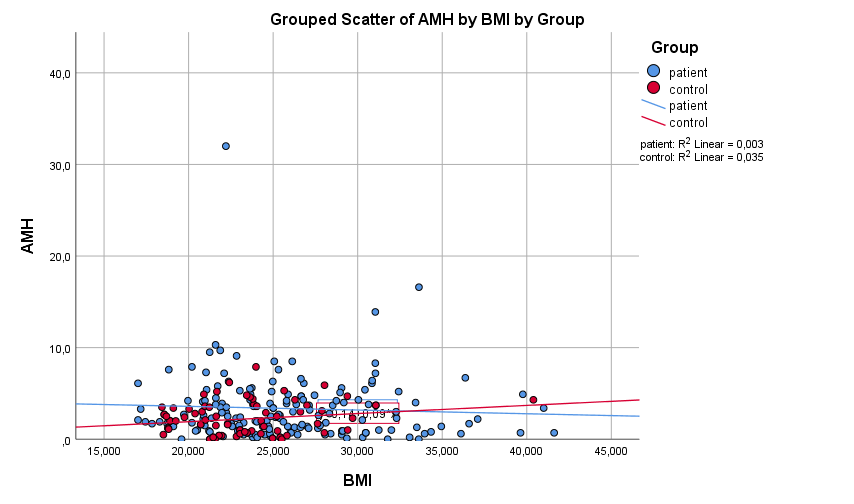
**Appendix 1. Correlation between AMH and age, blood pressure and BMI**

Supplement: Supplementary file 1 — Supplementary Material 1 [file 13048_2023_1333_MOESM1_ESM.docx]
